# Sealing effectiveness of fissure sealant bonded with universal adhesive systems on saliva-contaminated and noncontaminated enamel

**DOI:** 10.4317/jced.54471

**Published:** 2018-01-01

**Authors:** Mahtab Memarpour, Fereshteh Shafiei, Mehran Zarean, Faranak Razmjoei

**Affiliations:** 1 DMD, MScD, Professor, Oral and Dental Disease Research Center, Department of Pediatric Dentistry, School of Dentistry, Shiraz University of Medical Sciences, Shiraz, Iran; 2 DMD, MScD, Professor, Oral and Dental Disease Research Center, Department of Operative Dentistry, School of Dentistry, Shiraz University of Medical Sciences, Shiraz, Iran; 3 DDS, Dentist, Department of Pediatric Dentistry, School of Dentistry, Azad Shiraz University of Medical Sciences, Shiraz, Iran; 4 DMD, MScD, Assistant Professor, Oral and Dental Disease Research Center, Department of Pediatric Dentistry, School of Dentistry, Shiraz University of Medical Sciences, Shiraz, Iran

## Abstract

**Background:**

The effectiveness of sealants is dependent upon their adhesion to enamel surface. The aim of the study was to evaluate the sealing ability of a pit and fissure sealant used with a universal adhesive (etch-and-rinse vs. self-etch modes) when the site is contaminated with saliva. Adhesive properties were evaluated as microleakage and scanning electron microscopic (SEM) characteristics.

**Material and Methods:**

A total of 72 mandibular third molars were randomly divided into 6 groups (n=12). Occlusal pits and fissures were sealed with an unfilled resin fissure sealant (FS) material with or without saliva contamination. The groups included: 1) phosphoric acid etching + FS (control), 2) phosphoric acid etching + Scotchbond Universal (etch-and-rinse) + FS, 3) phosphoric acid etching + saliva + Scotchbond Universal (etch-and-rinse) + FS, 4) Scotchbond Universal (self-etching) + FS,5) Scotchbond Universal (self-etching) + saliva + FS, and 6) Scotchbond Universal (self-etching) + saliva + Scotchbond Universal + FS. After thermocycling, the teeth were placed in 0.5% fuchsin, sectioned, and evaluated by digital microscopy. Two samples from each group were also observed by SEM. The data were analyzed with Kruskal-Wallis and Mann-Whitney tests for a significance of *p*<0.05.

**Results:**

There were significant differences among groups. Groups 1,2 and 4 showed the least microleakage, with no significant differences among groups. Saliva contamination led to increased microleakage and gap formation in SEM images in groups 3, 5 and 6.

**Conclusions:**

The fissure sealing ability of the universal adhesive in etch-and-rinse or self-etch modes was similar to that of conventional acid etching. Saliva contamination had a negative effect on sealant adhesion to pretreated enamel.

** Key words:**Pit and fissure sealant, Universal adhesive, Saliva.

## Introduction

Pit and fissure sealing is a widely accepted method to prevent dental caries on occlusal surfaces, especially in newly erupted permanent teeth in children (1). However, sealant application in young patients is a challenge for dentists due to difficult access to the teeth, the possibility of saliva contamination, and sometimes lack of cooperation by the patient (1,2).

Conventional methods of sealant therapy include enamel acid etching (followed by a rinsing step to remove dissolved minerals) to create microporosities that facilitate penetration of the sealant material in the enamel (3). Because the formulation of resin sealant is similar to composite resin, some studies reported that adding an adhesive layer beneath the fissure sealant led to increased retention and reduced mircoleakge through the sealant and enamel surface (4,5). However, other studies found that the addition of this step had no advantages (6,7). One study reported that applying an adhesive reduced the negative effect of saliva contamination on sealant bond strength to the enamel surface (8).

In general, adhesives are categorized as total-etch (two- or three-step adhesives) or self-etch (one- or two-bottle adhesives). Recently a new generation of one-bottle adhesives called “universal”, “multi-mode” or “multi-purpose” adhesives was developed. Universal adhesives (UA) have advantages compared to previous generations: they can be used in two modes, i.e., either as an etch-and-rinse adhesive with selective acid etching before application of the adhesive, or as a one-bottle self-etching adhesive without additional etching. Moreover, UAs are simpler, more user-friendly and less technique-sensitive, and reduce patient chair time – all of which are advantages in pediatric dentistry (9-12). Some studies showed that acid etch techniques had advantages over self-etch adhesives before sealant therapy (13,14). However, other studies found no significant differences in terms of microleakage between conventional acid etching techniques and self-etch adhesives before sealant application (15,16). One study showed that UA in etch-and-rinse mode yielded sealing outcomes similar to the self-etch mode of fissure sealant application (17).

Because few data are available on the use of UAs in sealant therapy, the purpose of this in vitro study was to determine the sealing effectiveness of a new one-step self-etch adhesive. The adhesive was applied with two different methods: selective pre-etching (etch-and-rinse mode) and self-etching mode, with or without saliva contamination. The results were compared by measuring microleakage and observing morphological characteristics of the tooth–sealant interface with scanning electron microscopy (SEM).

## Material and Methods

After approval of the research protocol by the Human Ethics Review Committee of the School of Dentistry, Shiraz University of Medical Sciences, 72 sound mandibular third molars were cleaned with a prophylaxis brush and disinfected by immersion in 0.1% chloramine T solution for 2 months. The teeth were assessed to discard those with defects, cracks or caries.

The samples were randomly divided into 6 groups of 12 teeth each, in which pretreatment steps were performed on occlusal surfaces before fissure sealant (FS) application as follows.

Group 1. Phosphoric acid etch + FS (control group) 

Group 2. Phosphoric acid etching + Scotchbond Universal (SBU) (etch-and-rinse mode) + FS 

Group 3. Phosphoric acid etching + saliva + SBU (etch-and-rinse mode) + FS

Group 4.SBU (self-etch mode) + FS

Group 5.SBU (self-etch mode) + saliva + FS

Group 6. SBU (self-etch mode) + saliva + SBU + FS

The adhesive was applied according to the manufacturer’s instructions ( Table 1).

**Table 1:**
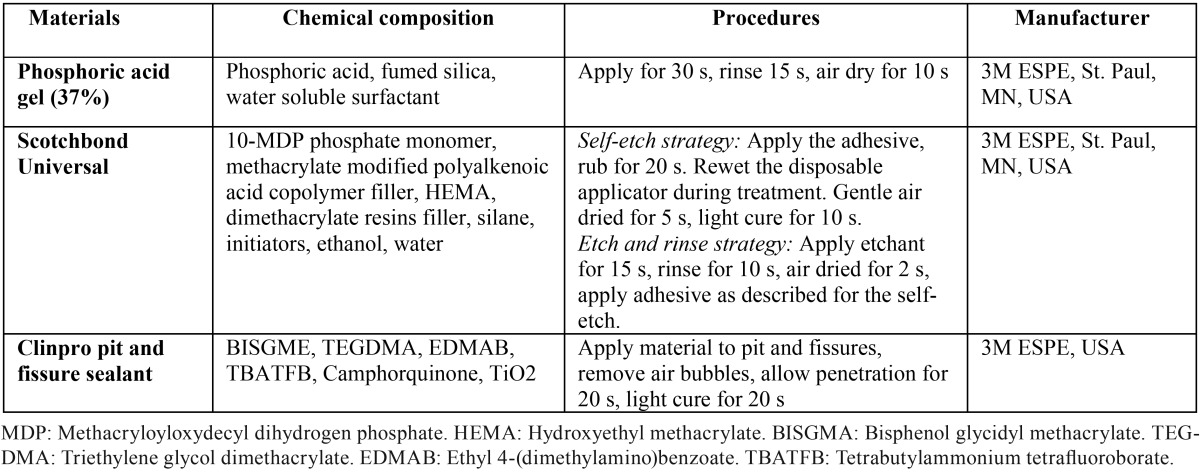
Materials and application procedures used in this study.

Saliva collection: The method used to collect saliva was based on a previous study (18). Unstimulated human saliva was collected from a 7-year-old child1hour after food or drink consumption. “The sample was centrifuged and stored at −80 °C. The saliva was thawed at room temperature and about 6 µL of saliva was applied on the tooth surface with a micropipette, left undisturbed for 10 s, and air dried for 5 s”. Then the adhesive and FS were applied.

Fissure sealant application: The occlusal surfaces were sealed with unfilled fissure sealant (Clinpro, 3M ESPE, St. Paul, MN, USA) and light-cured for 20 s with a halogen light curing unit (Coltolux, Coltene, Whaledent, Altstaetten, Switzerland) at a power density of 550 mW/cm2.

The specimens were then aged in a thermocycling bath at temperatures between 5 °C and 55 °C for 1000 cycles. The root apices were sealed with sticky wax, and the entire the tooth surface except for 1mm around the margins of each fissure sealant was covered with two layers of nail polish. Then the specimens were immersed in 0.5% basic fuchsin dye (Merck, Darmstadt, Germany) solution for 24 h to observe microleakage. Next the samples were rinsed and sectioned faciolingually across the center of the sealant with a diamond saw (Mecatome, Presi, Eybens, France) under continuous water irrigation.

Two dentists observed the sectioned teeth under a digital microscope (Dino Lite, Taipei, Taiwan) at 50× magnificationto score linear dye penetration in millimeters from the margin of the fissure sealant through the interface between the tooth and sealant. The proportion of microleakage was calculated by dividing the total distance of dye penetration by the total length of the enamel sealant interface (19). The microscope was calibrated before evaluations. To ensure inter observer agreement, the examiners evaluated 10 sectioned teeth before actual test samples were evaluated. These 10 teeth were not included in the sample used for analysis.

SEM observation: Two samples in each experimental group were selected for SEM evaluation. The specimens were sectioned perpendicular to the adhesive interface and polished with 400, 600, 1000 and 2000 grit silicon carbide paper under water cooling. Then the tooth surface was treated with 37% phosphoric acid for 10 s, rinsed for 30 s, and immersed in 5% NaOCl for 2 min. After rinsing, the teeth were dehydrated in a series of 70%, 80%, 90% and 99% ethanol. Next the samples were coated with gold in a vacuum evaporator, and the interfaces were examined in a SEM (KYKY-EM3200, Beijing, China) at 1000×magnification.

Statistical analysis: The data are reported as the median, mean rank and interquartile range (IQR). The assumption of normality was tested with the Shapiro–Wilk test. Kruskal–Wallis H and Dunnpost-hoc tests were used to compare microleakage among groups. Statistical analyses were done with IBM SPSS version 22.0 statistics software (IBM, New York, NY, USA). For all comparisons, *p* values less than 0.05 were considered statistically significant.

## Results

Shapiro–Wilk tests indicated that the data were not normally distributed in most groups.  Table 2 summarizes microleakage for all groups. Overall, we found significant differences among groups (*p*<0.001). The results of pairwise comparisons ( Table 3) indicated that microleakage was significantly lower in groups 1 (median=0, IQR=0.09) and 2 (median=0, IQR=0.34) compared to groups 3 (median=0.52, IQR=0.19), 5 (median=0.67, IQR=0.37), and 6 (median=0.66, IQR=0.16) (all *p*<0.05). However, there were no significant differences between other groups.

**Table 2:**
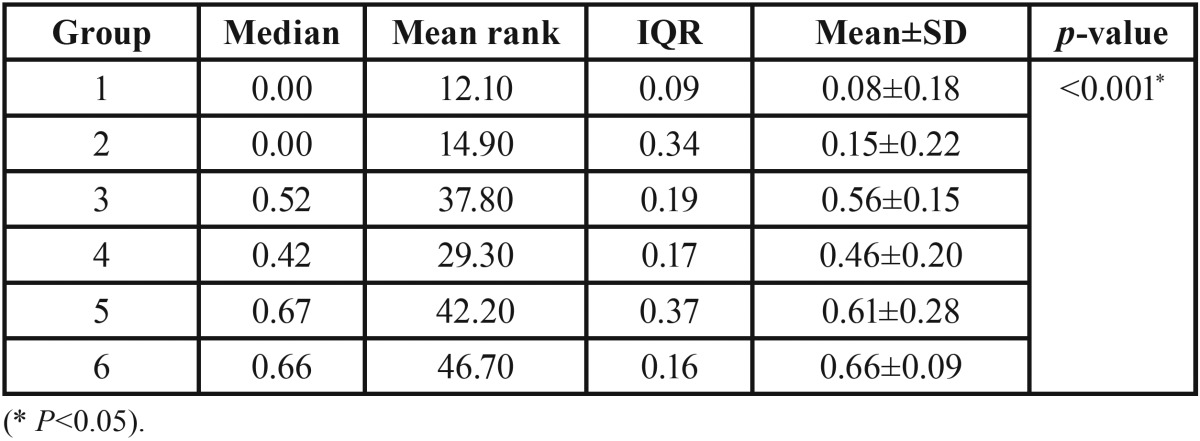
The description of microleakage for the groups (n=12).

**Table 3:**
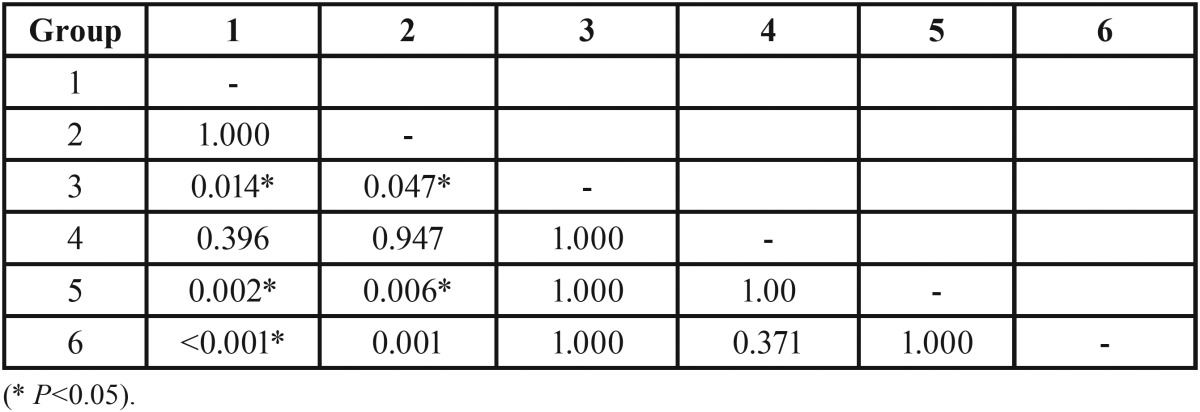
The results of pairwise comparisons.

**Figure 1:**
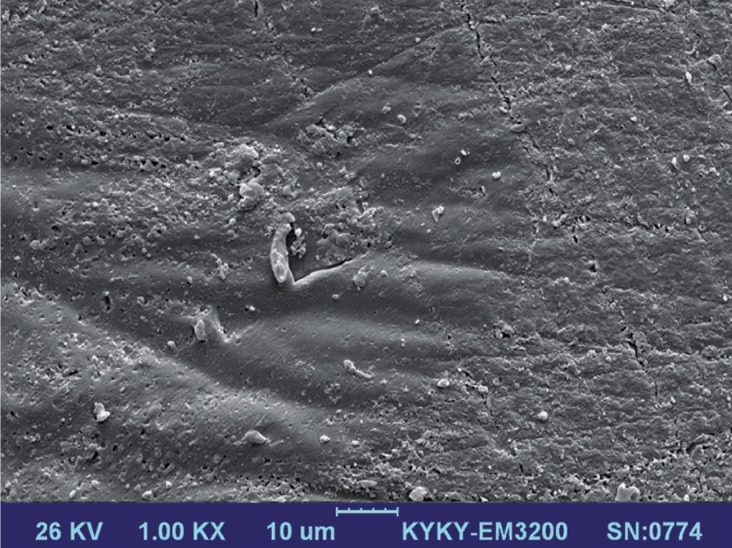
SEM image of the tooth–fissure sealant interface when SBU was applied in etch-and-rinse mode.

**Figure 2:**
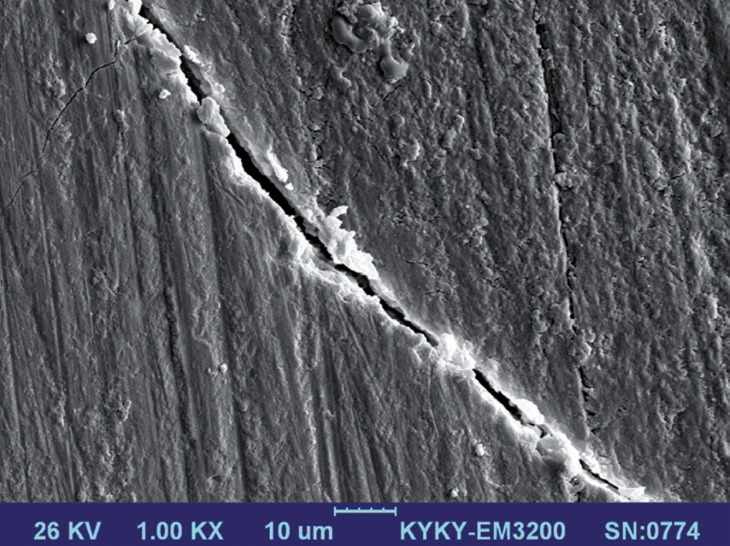
SEM image of the tooth–fissure sealant interface when SBU was applied in self-etch mode.

**Figure 3:**
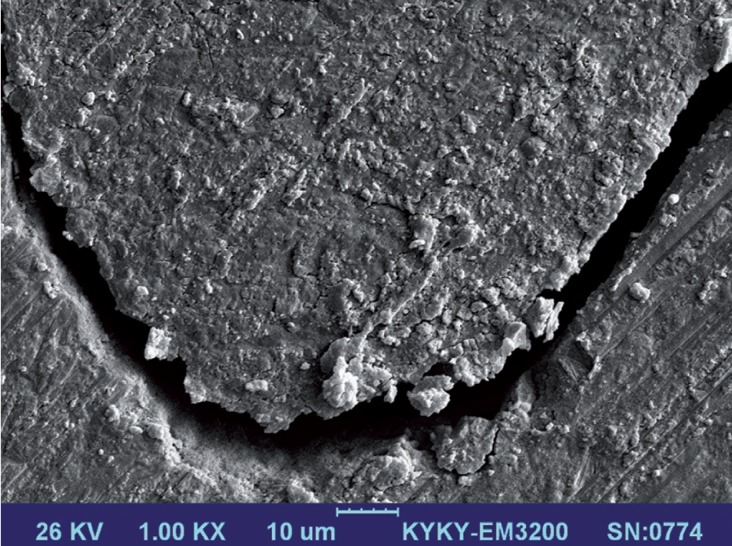
SEM image of the tooth–fissure sealant interface when SBU was used in the presence of saliva contamination.

The SEM images of all groups were assessed at the FS and enamel interfaces. Group 1 and 2 interfaces showed resin penetration into the enamel in the deepest points and lateral walls (Fig. 1). The application of SBU in self-etch mode (group 2) resulted in less demineralization and slight infiltration compared to SBU in etch-and-rinse mode (group 1) (Fig. 2). Saliva contamination prevented interlocking along the enamel–sealant interfaces, and the formation of gaps along the fissure sealant and lateral walls of the tooth surfaces. In addition, pooling of the saliva in the deepest part of the fissure led to frank gap formation in groups 3,5,6 (Fig. 3).

## Discussion

This study compared microleakage and SEM images of the FS–tooth interface in permanent teeth after the application of UA in etch-and-rinse and self-etch modes with or without saliva contamination. As in previous studies, all experimental groups in the present study showed some degree of microleakage (7,16). We found no significant differences in the extent of microleakage between the use of UA in etch-and-rinse or self-etch modes, or between either of these groups and the control group. This finding is consistent with a previous study that reported no difference in the sealing ability of SBU used in self-etch or etch-and-rinse mode (17). For pediatric patients, the self-etch technique as potential advantages especially in sealant therapy, because clinical procedures are simpler and technique sensitivity is lower (11,12).

The bond strength of an adhesive is related to its enamel etching capacity as well as its mechanical properties (20). Scotchbond Universal, used in the present study, contains the acidic monomer10-methacryloyloxydecyl (10-MDP), which interacts chemically with calcium in the hydroxyapatite present in enamel. However, some reports have noted that UA is less acidic than phosphoric acid (10-12). Some researchers have therefore recommended selective pre-etching to increase bond strength between the composite and enamel (21).

We used a low-viscosity resin-based sealant that performed well in dry conditions. It penetrated to deep parts of tooth fissures, and adapted well to the pit and fissure walls, as noted in a previous study (7,22). However, adequate moisture control may not be possible during sealant therapy, especially in young children. Therefore an adhesive amenable to simpler application techniques, along with precautions to reduce saliva contamination, should be considered (18). Although SBU contains VitrebondTM, a copolymer which is resistant to moisture (23), our results in teeth treated with SBU showed that the presence of saliva increased microleakage and influenced penetration of the resin to the deep parts of the fissures. In this connection, some studies reported the presence of a smear layer, and noted that contamination by moisture or saliva resulted in decreased adhesion of the sealant to the enamel (22,24). These effects may be due to the partial occlusion by saliva of some microprosityspaces (25). In contrast, Santschi *et al.* found that saliva contamination did not influence the shear bond strength of SBU in dentin compared to Xeno V (18). Peng *et al.* reported that microleakage increased when the enamel was contaminated with dried saliva before Fuji Triage sealant was applied (26). In contrast, another study demonstrated that contamination by dried saliva had no negative effect on the penetration or adaptation of a resin-based fissure sealant (27). In the present study we used saliva contamination to simulate conditions that may occur during sealant therapy. Our findings were consistent with earlier reports that in wet conditions, i.e. in the presence of water or saliva without drying, sealant penetration and adaption are compromised (22,25). The differences in the results among studies may be related to the different types of tooth substrate or adhesive used, and to differences in the methods used to decontaminate tooth surfaces (18).

We used SEM images to observe sealant adaption to and penetration of enamel surfaces after different enamel pretreatments before FS was applied. Conventional acid etching as well as UA either in etch-and-rinse or self-etch modes resulted in adaptation of the sealant to the lateral walls of the teeth. However, saliva led to gaps in the interface, as observed in our assessment of microleakage. One study of a resin-based sealant material reported that it demonstrated less adaptation and penetration under wet conditions than in dry conditions (22).

Laboratory testing for microleakage is a useful method to assess sealing ability. However, in vitro studies may not reflect clinical conditions accurately. Microleakage along the interface between the tooth surface and restorative material may result in recurrent caries (28). Therefore further clinical studies should be designed to evaluate the efficacy of SBU as a fissure sealant in children.

## Conclusions

Our results showed no significant difference in the extent of microleakage between conventional acid etching and application of a universal adhesive either in etch–and-rinse or self-etch modes before the application of a resin-based fissure sealant. The universal adhesive tested here may offer an alternative method for enamel surface pretreatment before sealant application. Saliva contamination had a negative effect on sealing ability, even when an adhesive step was added.
